# Presence and origin of variability of the pull test and push-and-release test in people with Parkinson’s disease

**DOI:** 10.1007/s00415-025-12974-9

**Published:** 2025-03-08

**Authors:** Jamie A. F. Jansen, Bastiaan R. Bloem, Noël Keijsers, Jorik Nonnekes, Vivian Weerdesteyn

**Affiliations:** 1 Donders Institute for Brain, Cognition and Behavior, Department of Rehabilitation, Center of Expertise for Parkinson & Movement Disorders, Radboud University Medical Center, Nijmegen, The Netherlands; 2 Donders Institute for Brain, Cognition and Behaviour, Department of Neurology, Centre of Expertise for Parkinson & Movement Disorders, Radboud University Medical Centre, Nijmegen, The Netherlands; 3 Department of Research, Sint Maartenskliniek, Nijmegen, The Netherlands; 4 Donders Institute for Brain, Cognition and Behaviour, Department of Sensorimotor Neuroscience, Radboud University, Nijmegen, The Netherlands; 5 Department of Rehabilitation, Sint Maartenskliniek, Ubbergen, The Netherlands

**Keywords:** Postural instability, Parkinson’s disease, Pull test, Push-and-release test, Balance control, Balance perturbation

## Abstract

**Background:**

The pull test and the push-and-release test evaluate postural instability in Parkinson’s disease (PD). We systematically evaluated the impact of within- and between-assessor variability in test delivery by the clinician on the test outcome. We also evaluated whether using standardized treadmill-based mechanical perturbations may enhance the consistency of the patient’s test outcomes.

**Methods:**

Fifty persons with PD underwent a series of backward balance perturbations: three repetitions of both the pull test and the push-and-release test delivered by three different assessors (i.e., nine repetitions of each test), plus five standardized treadmill-induced perturbations at 1.5 m/s^2^, in pseudo-random order.

**Results:**

We found substantial within-assessor variability on both manual tests. A difference in scores of 2 points or more was found in 30% of participants for the pull tests, and in 42% for the push-and-release tests. Similarly, large variability in scores was observed between assessors. Inconsistent test delivery was demonstrated by a wide range of sternum and center of mass displacements following the pull test and body inclination angles in the push-and-release test. Across five repeated treadmill-based perturbations at 1.5 m/s^2^, ≥ 2 points difference in test outcomes was found in 18% of participants, with significantly greater consistency in sternum and center of mass displacements.

**Conclusions:**

Variability in the patient’s balance test scores can be attributed to substantial variability in test delivery, as well as inconsistent performance of the individual patient. Assessment of postural instability may benefit from standardizing test delivery, e.g., using treadmill-induced perturbations.

## Introduction

Postural instability is one of the cardinal signs in persons with moderate to severe Parkinson’s disease (PD). It leads to falls and fall-related injuries, and negatively impacts on mobility, quality of life, and independence [1–3]. Valid assessment of postural instability is key to optimize patient management, observe treatment efficacy, and monitor disease progression. Yet, a study that evaluated disease progression data from three clinical trials found that more than half of the patients in whom an onset of postural instability had been clinically identified reverted to normal at a subsequent visit [4]. This inconsistency suggests potential flaws in the application of clinical tests for identifying postural instability, raising concerns about their reliability.

In daily practice, two tests are commonly used and regarded as the gold standard to evaluate postural instability [5]. The ‘Pull test’, part of the Movement Disorders Society Unified Parkinson Disease Rating Scale (MDS-UPDRS), examines a person's reaction to a sudden pull on the shoulders strong enough to induce a backward loss of balance, forcing the person to take at least one step backward [6]. When using the ‘Push-and-release test,’ a backward loss of balance is induced by a sudden release of the examiner’s hands placed on the person's back who is actively applying a backward force [7]. With both tests, the absence or the number of balance-correcting steps taken indicates the degree of postural instability on a five-point ordinal scale.

Although it is a common clinical observation that the test outcome following both the pull and the push-and-release test can vary between clinicians and between subsequent perturbations within the same participant, remarkably few studies have formally evaluated the consistency in test delivery by therapists in persons with PD. One study in people with PD demonstrated that errors in pull test delivery (e.g., pull intensity, ‘if the pull was administered at the shoulders’, or ‘whether the pull was performed steady and continuously’) were highly prevalent among a group of 25 assessors, resulting in almost 80% of the pull tests conducted being unsuitable for adequately assessing postural instability. In addition, one study observed moderate-to-low inter-rater reliability when examining variability in test delivery between assessors in single patients, but somewhat surprisingly, this variability in test delivery did not influence test performance[8].Another study in a mixed population reported differences in the actual test outcomes across the ten repetitions within the same assessor in four of the 12 PD participants (33%), with small but significant effects on the participants’ responses in step time and length [9]. Furthermore, recurrent clinical evaluations of postural instability in persons with PD are commonly conducted by different health professionals. A study in healthy individuals reported poor to moderate consistency between assessors in push-and-release test delivery (i.e., initial body lean angle) [10]. Surprisingly, the impact of between-assessor test delivery variability on test performance has not yet been evaluated in persons with PD.

We here aimed to systematically evaluate the impact of within and between-assessor variability in pull test and the push-and-release test delivery on postural instability test outcome (i.e., test scores according to respective rating scale) in persons with PD. To this aim, we used a comprehensive testing protocol in 50 participants with PD (Hoehn and Yahr stages 1–3), with three assessors each administering three repetitions of both the pull and the push-and-release test. To study variability in test delivery by the therapists, we quantified the magnitude of the perturbation by assessing the sternum displacement in the pull test and the initial body lean angle in the push-and-release test at perturbation onset. We expected to observe variability in retropulsion performance in both the pull test and the push-and-release test within people with PD, in parallel with substantial variability in test delivery, more so between than within assessors.

**Fig. 1:**
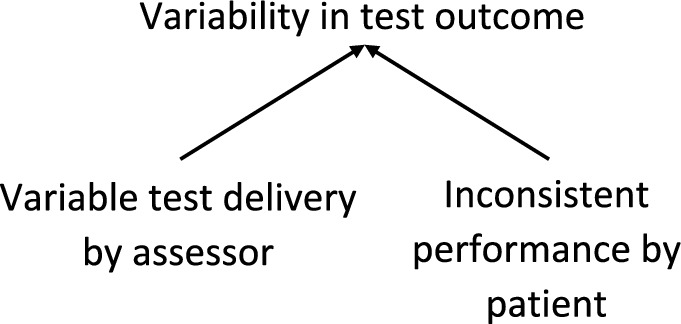
Variability in the patient’s test outcomes is determined by variable test delivery within and between assessors, as well as inconsistent performance of the patient him/herself

In addition, we evaluated variability in test outcomes following standardized treadmill-based mechanical perturbations. Recent work has demonstrated that using standardized treadmill-based perturbations may outperform the pull test in differentiating between persons with PD and healthy controls, possibly due to reduced test delivery variability [11]. We expected such standardized test delivery to be reflected in greater consistency in imposed body displacements, and consequently, lower variability in test outcomes than in the manual tests. Nonetheless, we expected to find residual variability in test outcomes as these are not only determined by variability in test delivery, but also by the consistency of the patient ‘s performance (Fig. 1).

## Methods

### Participants

In this observational cohort study, we included 50 people with PD (defined according to accepted international criteria), in the dopaminergic ON state, who were able to stand unaided (Table 1). Persons with PD were excluded if any other neurological, musculoskeletal, or orthopedic condition affected balance capacity, or if people were unable to follow instructions. This study was conducted in accordance with the Declaration of Helsinki [10] and with approval of the local ethical committee (Medical Ethical Committee Arnhem-Nijmegen dossier ‘2021–13298 ‘). All participants provided written informed consent before participating.

**Table 1:** Population characteristics

Age (years)	67 ± 7
Men (*n*, %)	25 (50%)
Time since diagnosis (years)	6.5 ± 4.7
MDS-UPDRS part III score (median, [0–136])	32.5 [4–72]
Hoehn and Yahr stage (median, [1–5])	2 [1–3]
Fallen in the past year (*n*, %)	21 (42%)
More than one fall in the past year (*n*, %)	14 (28%)
Subjective presence of freezing of gait^a^ (*n*, %)	12 (24%)
Mini-BESTest score (median, [0–28])	23 [10–28]
MoCA score (median, [0–30])	28 [22–30]

Values are represented as mean ± SD, unless otherwise specified

^a^Defined by a score of > 0 on the NFOG-Q

### Experimental protocol

Measurements took place at the Radboud University Medical Center (Radboudumc, Nijmegen, the Netherlands). Information on demographics, disease duration, and fall history was collected. Disease severity was assessed using the MDS-UPDRS part III [6]. Subjective presence of freezing of gait was identified using the New Freezing of Gait Questionnaire (NFOG-Q) [12]. Cognitive assessment involved the Montreal Cognitive Assessment (MoCA) [13]. Furthermore, balance capacity was clinically evaluated using the mini-BESTest [14].

**Fig. 2:**
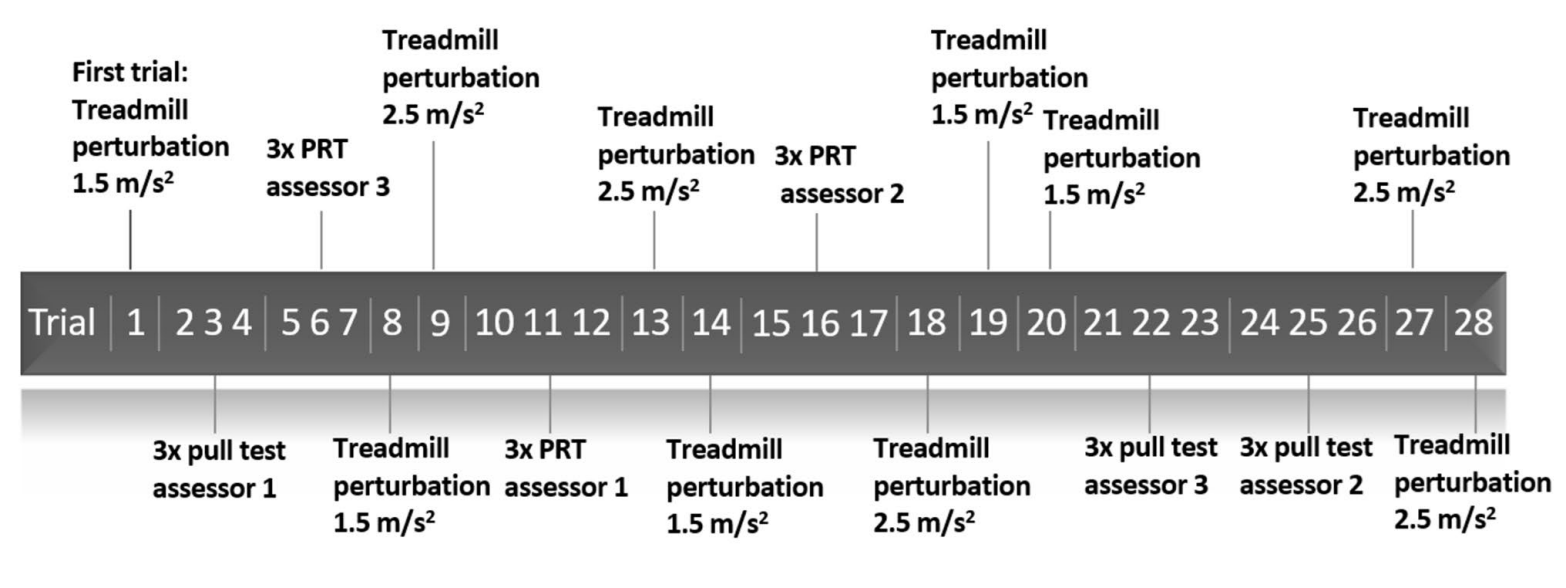
Example of a pseudo-randomized trial sequence for a participant. The first trial was always a treadmill perturbation at 1.5 m/s^2^. PRT: Push & release test

Participants stood on the surface of an instrumented dual-belt treadmill (Motek, Amsterdam, NL). To prevent falls and injuries, participants wore a safety harness attached to the ceiling. Safety bars were attached to the treadmill, but participants were instructed not to use these unless necessary. In total, subjects underwent a series of 28 trials involving backward balance perturbations. These included three times the pull test by three different trained assessors with similar experience (nine pull tests in total), three times the push-and-release test by three different assessors (nine push-and-release tests in total), five times a standardized treadmill-induced balance perturbation at 1.5 m/s^2^, and five times at 2.5 m/s^2^ (Fig. 2). The perturbation waveform comprised an acceleration phase of 300 ms, a constant velocity phase of 700 ms, and a deceleration phase of 300 ms. Perturbations delivered at 2.5 m/s^2^ were not included in the analyses since this intensity clearly exceeded those of the pull and push-and-release tests. The *first trial* always involved a treadmill-induced balance perturbation at 1.5 m/s^2^. Subsequent trials were conducted in a pseudo-randomized order that was different across participants to balance general habituation effects across perturbation types. The assessors always delivered three consecutive trials of either the pull test or the push-and-release test. Assessors never saw another perform either test before executing the test themselves. All trials were videotaped.

The pull test was conducted according to the instructions of the MDS-UPDRS part III, item 12 [6]. Before each pull test, the participants were instructed to stand upright with their feet comfortably wide and parallel next to each other. In addition, the person was allowed to take a step backward to prevent a fall. The assessors executed the pull test by applying a quick and forceful pull to the shoulders, forceful enough to displace the center of mass so that at least one step was needed to prevent a fall. Pull test trials were rated offline using the video-data and according to the ordinal scale of the MDS-UPDRS part III item 12 [6], i.e., (0) recovery with one or two steps; (1) three to five steps, but participant recovers unaided, (2) more than five steps but participant recovers unaided, (3) stands safely, but with absence of postural response; falls if not caught by assessor, (4) very unstable, tends to lose balance spontaneously or with just a gentle pull on the shoulders. The last did not occur in our study, as one of our inclusion criteria would interfere. Whenever a participant utilized the safety bars or used multiple steps but failed to recover balance before reaching the end of the treadmill surface and stepped into an assessor, the trial received a rating of 3.

The push-and-release test was conducted following the respective test instruction [7]. Before each trial, participants were instructed to stand upright with their feet comfortably wide and parallel. Participants were given instructions to do whatever was required to regain balance, including taking a step backward. After the assessor placed their palms on the participant’s scapulae, the participant was instructed to push backward. Flexion of the assessor’s elbows was required to allow backward movement of the participant’s trunk. Assessors released the participant when their shoulders and hips were behind the participant’s heels. Push-and-release test trials were rated using the push-and-release ordinal rating scale [7], i.e., (0) recovers independently with 1 step, (1) two or three backward steps but recovers unaided, (2) four or more backward steps, but recovers unaided, (3) steps backward, but needs assistance to prevent a fall, 4) falls without taking a step or is unable to stand without aid. Whenever a participant used the safety bars or used multiple steps but failed to recover balance before reaching the end of the treadmill surface and stepped into an assessor, they received a rating of 3 or 4 (depending on whether a step was taken).

In the treadmill-induced balance perturbations, participants were instructed to stand upright with their feet comfortably wide and parallel next to each other. Participants were instructed to try to recover from the balance perturbation by taking one step backward. Treadmill-induced balance perturbation trials were rated using the ordinal scale of the MDS-UPDRS part III item 12 [6]. Whenever a participant used the safety bars or used the safety harness to regain balance, the trial received a rating of 3.

### Data acquisition

A 10-camera motion capture system (Vicon Motion System Ltd., Oxford, UK) was used to record 3D kinematics at a sampling rate of 100 Hz. Reflective markers were attached to the surface of the participant’s skin. We placed markers on the sternum, pelvis and the feet. To correct for the movement of the treadmill, an additional marker was placed on the treadmill’s belt. The raw data was preprocessed in Vicon Nexus (Vicon Motion Systems Ltd., Oxford, UK).

### Data analysis

Vicon data were analyzed using Matlab 2022a (MathWorks Inc., Natick, MA, USA). Data were filtered using a second-order zero-lag Butterworth filter with a cutoff frequency of 10 Hz. The marker at the top of the sternum was used to identify sternum displacement, whereas the center of the four pelvis markers was used to estimate the CoM. To identify perturbation onset in the pull test and push-and-release test, we first localized the peak acceleration of the sternum marker. We then determined the determined the start of the slope, with the assumption that the acceleration had to be a positive value. In the treadmill condition, the start of the perturbation was determined by the belt marker’s velocity exceeding a threshold of 0.1 m/s. The relative displacement of the sternum and CoM at 250 ms following perturbation onset was identified to determine the variability in delivery of the pull test and the treadmill perturbations. We used a marker to correct for the movement of the belt. This instant was chosen to minimize the influence of corrective torques generated by the participant on the observed CoM and sternum displacements [15]. Push-and-release test delivery was expressed using the body inclination angle at perturbation onset. This involved the angle of the sternum marker relative to the middle of the participant’s toe markers.

### Statistical analysis

Variability in the pull and push-and-release test outcomes is presented descriptively as (1) maximum within-participant difference in ratings across all nine trials; (2) difference in first trial and in median ratings *between* each assessor; (3) difference in ratings between three repetitions *within* each assessor. Variability in outcomes following standardized perturbations on the treadmill is presented as the maximum within-participant difference in outcome in ratings across five repetitions. As the very first trial of the protocol always involved a treadmill perturbation at 1.5 m/s^2^, which trial is known to yield distinctly different behavioral responses compared to any subsequent trial, we also present within-participant differences between trials two and five to eliminate this first-trial effect.

Variability in delivery of the pull test is descriptively presented as single-trial values of sternum and CoM displacements, as well as the observed range (1) across all nine repetitions within each participant; (2) across the first trials of each of the three assessors; and (3) across the three repetitions within each assessor. We used independent samples *T*-tests to test whether the range of sternum and CoM displacements across the nine trials differed from that of standardized treadmill perturbations. T-tests were also used to test whether the range in displacements *within* assessors differed from that *between* assessors. Similar statistics were used for comparing ranges in body inclination angles in the push-and-release test within and between assessors.

## Results

**Fig. 3:**
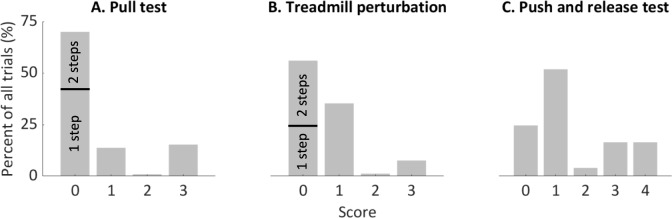
Distribution of ordinal scores across all recorded trials. **A** Scores of the pull test (*n* = 450) are rated according the MDS-UPDRS part 3.12. **B** Treadmill perturbations (*n* = 250) are rated according the MDS-UPDRS part 3.12. **C** The push-and-release test (*n* = 450) is rated according the ordinal scale used in the push-and-release test

Distributions of outcomes on the pull test, push-and-release test and treadmill perturbations across all participants are shown in Fig. 3. In all executed pull tests (*n* = 450), 70% of the pulls resulted in balance recovery with one (42%, *n* = 191) or two (27%, *n* = 123) steps (score 0, *n* = 314), 14% in three to five steps (score 1, *n* = 63), 1% in unaided recovery in five or more steps (score 2, *n* = 4), and 15% in an aided (i.e., by the assessor, handrails or the safety harness) recovery due to an absence of stepping response (score 3, *n* = 69). In 56% of the treadmill perturbations people recovered with one (26%, *n* = 64) or two (30%, *n* = 76) steps (score 0, *n* = 140), 35% with three to five steps (score 1, *n* = 88), 1% with five or more steps but unaided (score 2, *n* = 3), and 8% in an aided recovery (score 3, *n* = 19). In the push-and-release test, 25% of the people recovered with one step (score 0, *n* = 111), 52% of the trials resulted in two or three steps (score 1, *n* = 233), 4% in four or more step with unaided recovery (score 2, *n* = 18), 16% in backward steps with aided recovery (score 3, *n* = 74), and 3% resulted in an aided recovery without taking a step backward (score 4, *n* = 14).

**Fig. 4:**
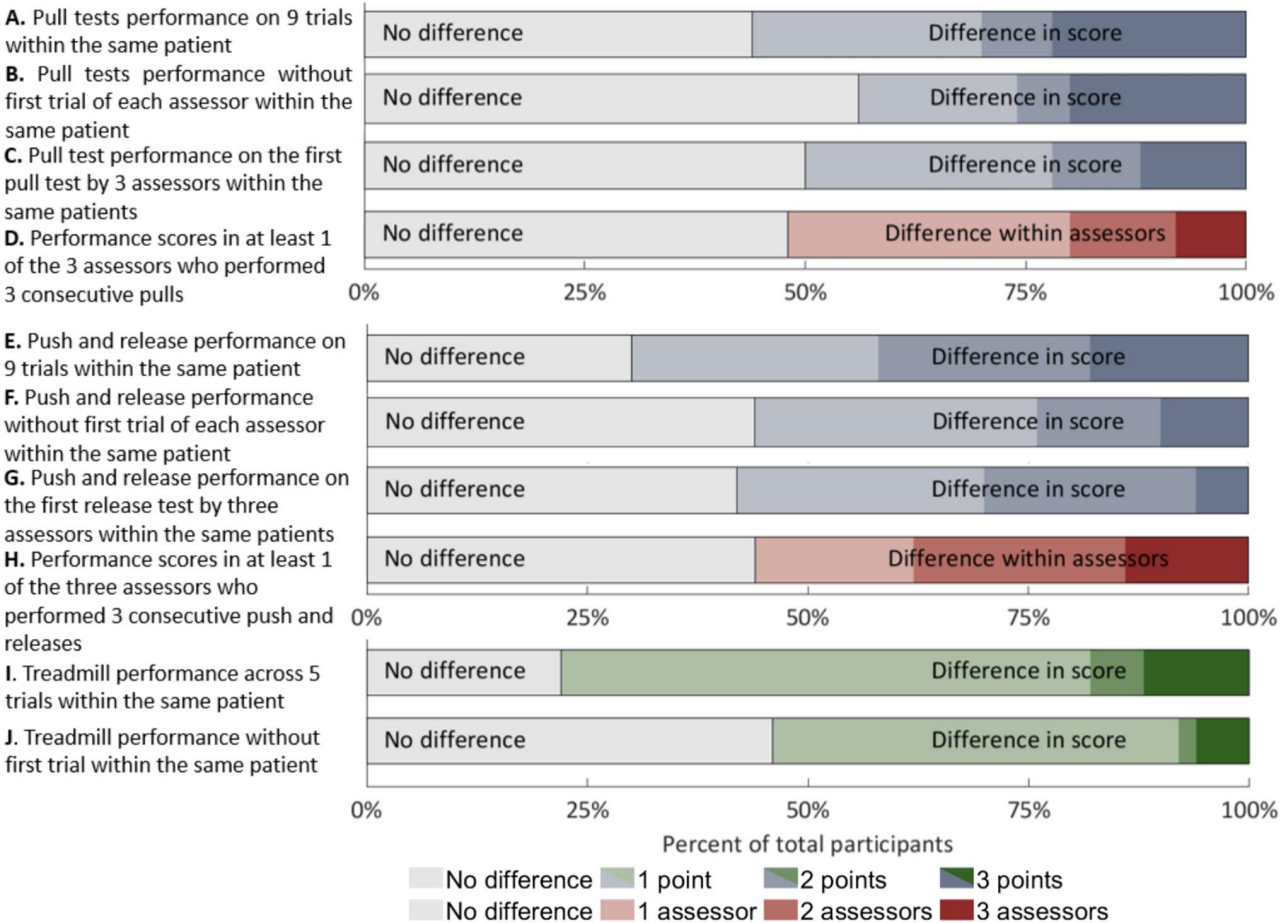
Variability within participants in performance on the pull test, the push-and-release test and the treadmill perturbations

Variability in outcome on the pull test, the push-and-release test, and treadmill perturbations is visualized in Fig. 4. Across nine pull tests of each participant, we observed variability in outcomes in 56% of the participants (*n* = 28), with differences in scores ranging from one point (26%) to three points (22%). When leaving out the first trial of each assessor, variability in outcomes was seen in 44% of participants, with differences in scores ranging from one point (18%) to three points (20%). Between the three assessors, the scores on their first pull showed variability in 50% of the participants (*n* = 25), whereas median pull test scores differed between the three assessors in 26% of participants (*n* = 13). Likewise, between the three consecutive pulls administered by the same assessor, 52% of the participants demonstrated variable scores in at least one of the three assessors (*n* = 26).

Across the nine push-and-release tests, we observed variability in outcomes in 70% of participants (*n* = 35), with differences in scores ranging from one point (28%) to three points (18%). When leaving out the first trial of each assessor, variability in outcomes was seen in 56% of participants, with differences in scores ranging from one point (32%) to three points (10%). When looking at the first push-and-release test of each of the three assessors, 58% of participants showed different scores between assessors (*n* = 29), whereas median scores differed in 44% of participants (*n* = 22). Within-assessor variability was observed in a comparable percentage of participants, with 56% showing different scores in three consecutive push-and-release tests of at least one of the three assessors (*n* = 28).

Seventy-eight percent of participants exhibited variability in outcomes across five treadmill-induced balance perturbations (*n* = 39). Yet, as expected, it was often the outcome in response to the first perturbation (i.e., always the very first trial of the experimental protocol) that differed from the other four trials. When discarding this very first trial, 54% of the participants showed variability in outcome scores (*n* = 27), with differences ranging from one point in 46% to three points in 6%.

**Fig. 5:**
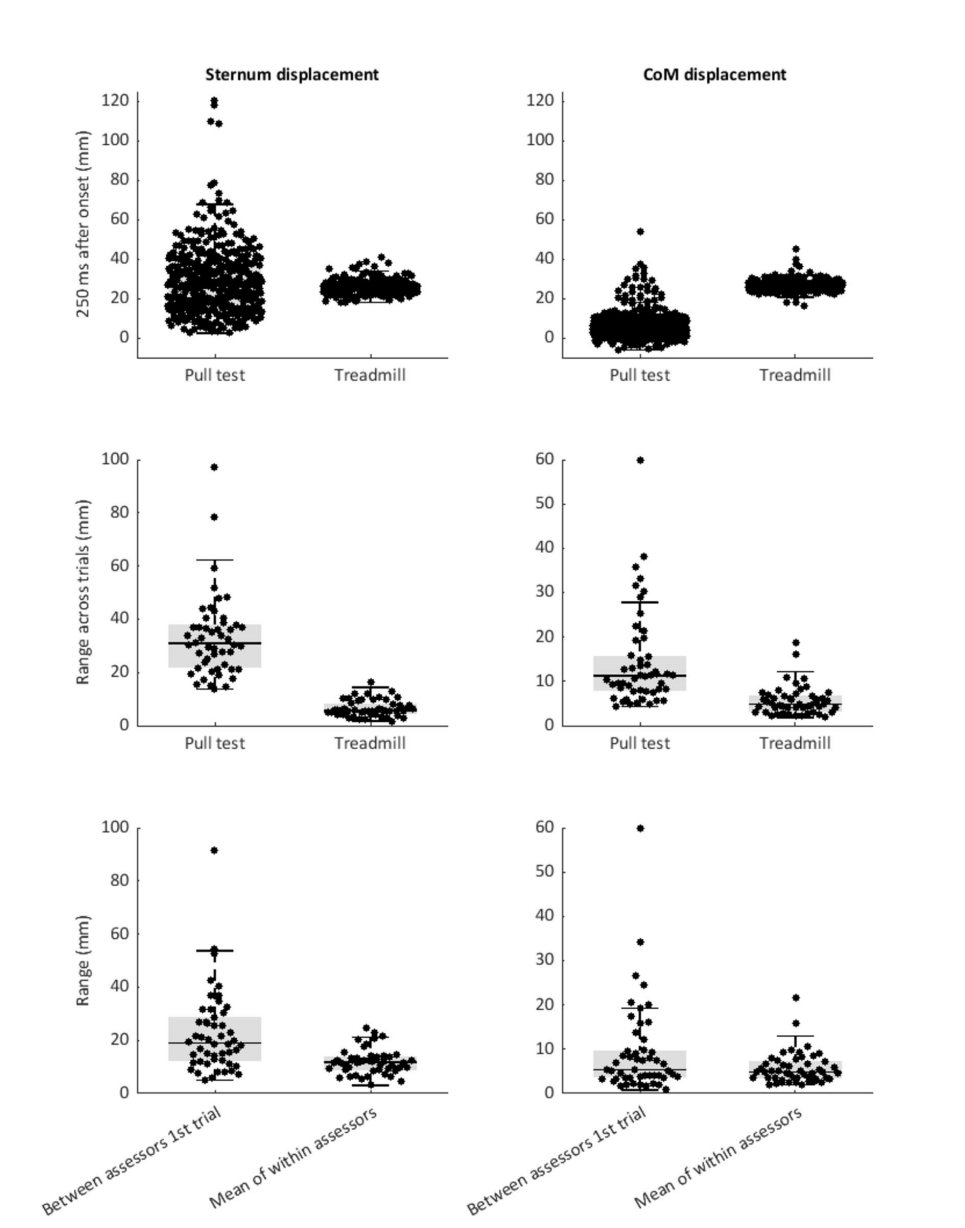
Sternum displacement and CoM displacement at 250 after pull onset, for the pull test and the treadmill perturbations. **A** and **B** represent all data points of the pull test and the treadmill perturbations. **C** and **D** represent ranges over 9 trials within the same patient. **E** and **F** depict the range in the first trial of each of the three assessors and the mean range within the same patient

Variability in the pull test delivery (i.e., the magnitude of the pull) is visualized in Fig. 5. Sternum displacement ranged from 0.2 to 12.1 cm in the pull test. While the range across all trials was evidently wider than following treadmill perturbations (1.8–4.1 cm), the average sternum displacements showed a minor 2 mm difference (2.8 ± 1.6 vs 2.6 ± 0.4 cm, respectively; *t*_(687)_ = 2.244, *p* = 0.025). Similarly, the range of CoM displacements was larger for the pull test (range: − 0.6 to 5.4 cm) compared to the treadmill condition (range: 1.6–4.5 cm), but average CoM displacements were smaller (0.6 ± 0.7 cm vs 2.6 ± 0.3 cm, respectively; *t*_(681)_ = 42.683, *p* =  < 0.001).

Across nine trials of the pull test within each patient, the range of sternum displacements varied between 1.4 and 9.7 cm (3.3 ± 1.5 cm), which was larger than the range observed within the five treadmill perturbations (0.2–1.7 cm; 0.7 ± 0.3 cm; *t*_(98)_ = 12.015, *p* < 0.001). For the CoM displacement, the range within each patient varied between 0.4 and 6.0 cm (1.4 ± 1.1 cm) in the pull test, which was larger than the range within the five treadmill perturbations (0.2–1.9 cm, 0.6 ± 0.3 cm; *t*_(98)_ = 5.499, *p* < 0.001).

Between each of the three assessors, the range of the sternum and CoM displacements in the first trials of the pull test (between-assessor variability in test delivery) varied between 0.5 and 9.2 cm (Mdn: 1.9 cm, IQR: 1.7 cm) and 0.1 and 6.0 cm (Mdn: 0.5 cm, IQR: 0.7 cm), respectively. The mean range of the sternum and CoM displacements within the same patient (within-assessor test delivery variability) varied from 0.3 to 2.5 cm (Mdn: 1.2 cm, IQR: 0.5) and 0.2 to 2.1 cm (Mdn: 0.5 cm, IQR: 0.4 cm), respectively. Between-assessor variability in sternum and CoM displacements was significantly larger compared to within-assessor variability (*t*_(98)_ = 21.707, *p* =  < 0.001 and *t*_(98)_ = 2.382, *p* = 0.019).

**Fig. 6:**
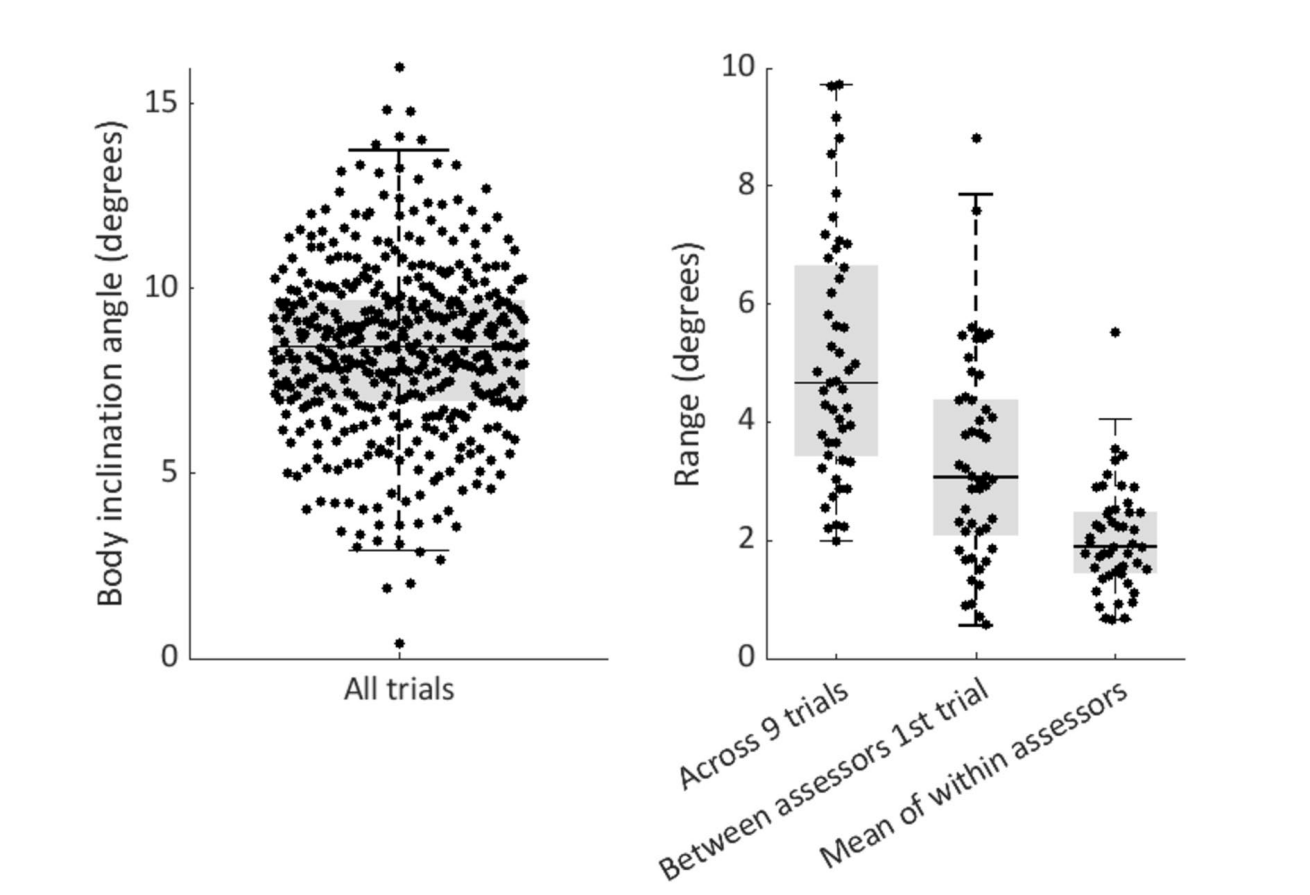
Push & release test: body inclination angles at perturbation onset. Variability across all data points and ranges across nine trials, the first three trials of each of the assessors and the mean range within each of the assessors

Variability in the push-and-release test delivery (i.e., the body inclination angle at perturbation onset) is visualized in Fig. 6. Body inclination angles across all trials ranged from 0.4 to 16.0 degrees (Mdn: 8.4 degrees, IQR: 2.7 degrees). The range of the body inclination angles across nine trials within the same patient varied between 2.0 and 9.7 degrees (Mdn: 4.6 degrees, IQR: 3.3 degrees). Between assessors, the range of the body inclination angle of the first push-and-release test (between-assessor test delivery variability) was 0.6–8.8 degrees (Mdn: 3.1 degrees, IQR: 2.3 degrees). When looking at the variability in test delivery within assessors, the mean range of the body inclination angles of three test repetitions within the same patient varied between 0.7 and 5.5 degrees (Mdn: 1.9 degrees, IQR: 1.0 degrees). Between-assessor variability was significantly larger compared to the within-assessor variability (*t*_(98)_ = 4.764, *p* =  < 0.001).

## Discussion

This observational cohort study in 50 persons with PD involves the first systematic evaluation of variability in postural instability test outcomes (i.e., test scores according to an established rating scale) within and between assessors. In line with our hypotheses, we found substantial variability in pull test and push-and-release test outcomes within participants. Across nine repetitions within each participant, a difference in pull test scores of 2 points or more was observed in 30% of participants, and in the push-and-release test scores in 42%. Variability in outcomes was rather similar within and between assessors. Across five repetitions of standardized treadmill-based perturbations at 1.5 m/s^2^, we observed a difference of 2 points or more in 18% of the participants. This, number further dropped to 8% when the very first trial was discarded, while the proportion of participants with consistent test scores (difference of 0 points) was comparable to that of the manual pull test (40–50%). Discarding the first trial of each assessor on the manual pull and the push-and-release tests also lowered the observed variability in test outcomes to some extent, but a difference of 2 points or more was still present in 26% and 24% of participants, respectively*.* We observed large variability in pull test and push-and-release test delivery, with sternum displacement and body inclination angles showing greater between- than within-assessor variability. Variability in sternum and CoM displacements was greatly reduced following standardized treadmill perturbations.

We are unaware of any previous systematic evaluation of *between*-assessor variability in retropulsion outcomes within the same participants with PD. In light of clinical experience, the presence of such variability may seem rather unsurprising, but the degree of variability in test outcomes was remarkably large. Variability in test scores on the pull test, as quantified using the ordinal scale on the MDS-UPDRS item 3.12, could be as large as 3 points within participants, indicating the difference between ‘no postural instability’ and ‘moderate postural instability’, with assistance required to recover from the backward balance perturbation. Variability in the push-and-release test scores was even more pronounced, which is likely due to differences in the ordinal rating scales of either test. In particular, recovery with two steps is considered ‘normal’ (score of 0) in the pull test, whereas this is regarded as mild postural instability (score of 1) in the push-and-release test. Indeed, as can be seen in Fig. 2, the relatively higher number of participants with a score of 0 in the pull as compared to the push-and-release test can largely be attributed to the participants who needed 2 steps to recover. These observations suggest that variability in pull test outcomes may have been underestimated due to the high proportion of participants in our study with a score of 0 in combination with the lack of sensitivity of the pull test in identifying mild postural instability. Collectively, these findings raise questions regarding the validity of postural instability assessments and warrant reconsideration of using these tests as a gold standard [4].

The finding of *within*-assessor variability in test outcomes is in line with a previous study in a small sample of PD participants (*n* = 12) [9]. In this study, one experienced physical therapist performed 10 backward push-and-release tests in each participant, with a difference in clinical rating scores observed in four participants (33%). As outlined in Fig. 1, variability in test delivery is proposed to at least partly explain the variability in test outcomes. Indeed, the present findings as well as the results from this previous study demonstrate that individual assessors appear to have difficulties calibrating their pull/ push-and-release for consistent test delivery, even when administering multiple repetitions within the same patient. Even when leaving out the first trial of each assessor, this takes away only a small part of the variability in test outcomes (Fig. 4). Unsurprisingly, when multiple assessors conducted these manual tests in the same patient, the observed greater ranges of imposed sternum and CoM displacements demonstrate that inconsistency in test delivery was further aggravated. These observations highlight the need for standardization of perturbation delivery, using e.g., pulley machines [16, 17], instrumented pull test [18, 19], and movable platforms [11, 20–24]). Our results show that delivering standardized treadmill-based balance perturbations substantially decreased the variability in imposed sternum and CoM displacements, and in line with our expectations, such standardization did take away some of the variability in test outcome. Yet, despite standardization, we observed residual variability in test outcomes, presumably due to inconsistencies in the participant’s test performance (Fig. 2).

The obvious disadvantages of treadmill-based perturbations are size and costs of the set-up, and thereby the application in daily clinical practice. As a more practical approach, wearable sensors and markerless video-based tracking methods have been proposed for quantifying manual pull intensity. Such metrics could potentially be used to provide assessors with feedback on their pull intensity and help standardize test delivery [8, 19, 25] However, identifying a parameter that gives a reliable readout is not straightforward due to two main reasons. First, pull intensity depends on multiple parameters, e.g., the energy delivered by the assessor is not only determined by the peak acceleration, but also by its duration. Second, because reactive responses of the participants commence soon after the onset of perturbation, metrics derived from movement trajectories of the participant’s body do not merely reflect the imposed perturbation, but are increasingly influenced by these responses. For the push-and-release test, such standardization methods may potentially be easier to implement, since perturbation intensity mainly depends on the inclination angle at the start of the perturbation. Yet, despite maximum standardization of test delivery on the treadmill, we still observed residual variability in test outcomes, presumably due to inconsistencies in the participant’s test performance (Fig. 1). Future work should establish the minimum number of repetitions needed for reliable quantification of postural instability. Interestingly, previous work from our group demonstrated that healthy older adults successfully recovered balance from standardized perturbations at 1.5 m/s^2^ in the vast majority of trials [24], whereas the PD participants in the present study managed to do so in only 26% of all trials. This observation indicates that postural instability was highly prevalent in our study population despite 70% of all pull tests yielding a score of 0 (i.e., no postural instability). In addition to the aforementioned rating differences (1 vs 2 steps), we speculate that some of the pulls may not have been strong enough to reveal postural instability. A previous study evaluated videotapes of pull test deliveries of 25 experienced assessors who performed a total of 66 pulls, which were evaluated by four raters with respect to pull technique and application (e.g., recovery space for the participant, feet placement of the patient, pull intensity, ‘if the pull was administered at the shoulders’, or ‘whether the pull was performed steady and continuously’) [26]. The most commonly agreed-on error (i.e., agreed by at least two of the four raters) was that the participant was pulled too lightly (*n* = 51, 77%). Indeed, the wide range of observed sternum displacements (Fig. 4) also included trials where the displacement at 250 ms after perturbation onset did not even exceed 1 cm. Such light pulls likely result in frequent false-negative test outcomes.

While standardized delivery of sufficiently strong perturbations holds promise in addressing important shortcomings of the currently used pull and push-and-release tests, assessment of postural instability may further benefit from quantification of step quality (in addition to the number of steps taken). To achieve this, one option to consider is measuring the leg angle (i.e., angle of the leg of the first balance-correcting step with the vertical at step touchdown), which measure captures the position of the foot relative to the CoM. Indeed, in response to standardized perturbations of equal magnitude, this metric was shown to distinguish between PD patients and controls [24], making it a potential candidate to evaluate postural instability on a continuous scale. Future studies may further explore the utility of the leg angle for quantifying postural instability in PD and for evaluating disease progression or effects of intervention.

It must be noted that center of mass displacements induced by the treadmill-induced perturbations differed from the pull tests. This is not surprising as the point of impact differs (i.e., the shoulder compared to the platform of the treadmill), and the duration of the impact is not the same. Despite these differences, the participants likely perceived test difficulty of the manual pull test and the treadmill perturbations as being rather similar as outcome scores were comparable between the tests (Fig. 2). A limitation of the study was that we performed a lower number of repetitions of the treadmill-based perturbations than pull and push-and-release tests. As a consequence, the variability in the treadmill-based tests may have been underestimated. Yet, it must be noted that variability in test outcome in four non-consecutively conducted treadmill-based perturbations (the very first trial excluded; see Fig. 2) was still somewhat lower than that of three consecutive pull or push-and-release test conducted by the same assessor. In addition, our protocol involved a series of relatively similar pseudo-randomized backward perturbations that may have led to habituation effects. This, however, may underestimate the variability in test outcomes observed in daily clinical practice, where retropulsion tests are typically performed longitudinally at multiple time points.

In conclusion, the present study demonstrates that an individual’s postural instability test outcomes may show substantial variability, presumably due to a combination of variable test delivery and inconsistencies in the patient’s performance. To rule out variability in test delivery, we recommend using standardized perturbations. To account for the observed variability in the patient’s performance, it seems imperative to administer multiple trials for accurately identifying postural instability.
